# Knowledge about Danger Signs of Obstetric Complications and Associated Factors among Postnatal Mothers of Mechekel District Health Centers, East Gojjam Zone, Northwest Ethiopia, 2014

**DOI:** 10.1155/2016/3495416

**Published:** 2016-06-08

**Authors:** Gedefa Amenu, Zerfu Mulaw, Tewodros Seyoum, Hinsermu Bayu

**Affiliations:** ^1^Department of Midwifery, College of Health Sciences, Dilla University, 419 Dilla, Ethiopia; ^2^Department of Midwifery, College of Health Sciences, University of Gondar, 196 Gondar, Ethiopia; ^3^Department of Midwifery, College of Health Sciences, Mekelle University, 231 Mek'ele, Ethiopia

## Abstract

*Background*. Developing countries like Ethiopia contributed highest level of maternal mortality due to obstetric complications. Women awareness of obstetric danger sign to recognize complications to seek medical care early is the first intervention in an effort to decrease maternal death.* Objective*. To assess knowledge about danger signs of obstetric complications and associated factors among postnatal mothers at Mechekel district health centers, East Gojjam zone, Northwest Ethiopia, 2014.* Methods*. An institution based cross-sectional study was conducted from August to October, 2014, in Mechekel district health centers. Systematic random sampling was used to select four hundred eleven study participants. A pretested structured questionnaire was used to collect data. Data were entered to Epi Info version 3.5.3 and exported to SPSS 20.0 for further analysis. Descriptive and summary statistics were done. Logistic regression analyses were used to see the association of different variables. Odds ratios and 95% confidence interval were computed to determine the presence and strength of association.* Results*. According to this study, 55.1% participants were knowledgeable about danger signs of obstetric complications. Maternal and husband educational level ((AOR = 1.977, 95% CI: 1.052, 3.716) and (AOR = 3.163, 95% CI: 1.860, 5.3770), resp.), family monthly income ≥ 1500 (AOR = 2.954, 95% CI: 1.289, 6.770), being multipara (AOR = 7.463, 95% CI: 1.301, 12.800), ANC follow-up during last pregnancy (AOR = 2.184, 95% CI: 1.137, 4.196), and place of last delivery (AOR = 1.955, 95% CI: 1.214, 3.150) were variables found to be significantly associated with women's knowledge on danger signs of obstetric complications.* Conclusion*. Significant proportion of respondents were not knowledgeable about obstetric danger signs and factors like educational status, place of last delivery, and antenatal follow-up were found to be associated.

## 1. Introduction

Pregnancy is a period when women's bodies go through serious physiological changes which may be entirely normal throughout pregnancy, childbirth, and postpartum period. However, this normal process may sometimes be overcome by serious complications which may affect the life of mothers and newborns contributing to maternal mortality and morbidity to the highest level [1].

World health organization (WHO) reported that, globally, an estimated number of 289,000 women died during and following pregnancy and childbirth related problem in 2013 alone, showing a decline of 45% from 1990 report. Developing countries like sub-Saharan (62%) and South Asia (24%) together contribute 86% of the problem [2, 3]. It has been reported that Ethiopia is one of the six countries that contributes about 50% maternal death, the others being India, Nigeria, Pakistan, Afghanistan, and the Democratic Republic of Congo [4, 5]. Despite government commitment to millennium development goal five (MDG_5_) to reduce maternal death by three-quarters over the period of 1990 to 2015, maternal mortality ratio (MMR) is still offtrack, estimated at 676 per 100,000 live-births in Ethiopia [6, 7].

Maternal mortality in resource poor nations has been attributed to three delays: delay in deciding to seek care, delay in reaching to seek care on time, and delay in receiving adequate treatment. Among all, the major cause for first delay is lack of awareness about obstetric danger signs to decide to seek care among mothers and community [8]. These danger signs are not the actual obstetric complications but symptoms that are easily identified by the mother herself and nonclinical personnel. They are danger signs like severe vaginal bleeding, severe headache, preterm labour, rupture of membrane before onset of labour epigastric pain, severe abdominal pain, prolonged labour (>12 hours), convulsions and retained placenta, foul-smelling vaginal discharge, and fever which probably occur during phases of pregnancy [9].

Women's knowledge about danger signs of obstetric complications is profoundly important to enhance utilization of skilled care during delivery and to seek emergency obstetric services. Lack of information on the warning signs of complications during pregnancy, parturition, and postpartum period hampers women's ability to partake fully in safe motherhood initiatives. As awareness of danger signs of obstetric complications is the essential first step in accepting appropriate and timely referral to obstetric care, it is vitally important that women and their families should have knowledge regarding danger signs of obstetric complications to enable them to respond appropriately [10].

National reproductive strategy of Ethiopia has given emphasis to raising mother's knowledge about danger signs of obstetric complications. According to this strategic plan by federal ministry of Ethiopia (FMOH), 80% of all families including mothers should recognize at least three danger signs associated with pregnancy related complications [11]. However, little is known about the current level of mothers' knowledge and associated factors in Ethiopia as research evidence from different parts of Ethiopia revealed [12, 13]. Therefore, this study aims to assess the current status of knowledge about obstetric danger signs and associate factors among postnatal mothers in the study area.

## 2. Methods

An institutional based cross-sectional study was conducted in Mechekel district health centers from August 1 to October 30, 2014, among postnatal mothers in six health centers. The district is located 328 km northeast of Addis Ababa, the capital city of Ethiopia. The potential study population comprised all postnatal mothers at Mechekel district health centers at the time of data collection irrespective of place of delivery.

Systematic sampling technique was used to select study participants at postnatal clinics of the six health centers at Mechekel district proportionally. The sample size was determined using a single population proportion formula considering the following assumptions: 58.8% proportion of knowledgeable women on danger signs and 5% level of significance (*α* = 0.005). The final sample size was adjusted for a nonresponse rate of 10% and the total sample arrived at was 411.

Data was collected by six diploma midwives through face to face interviews using a structured and pretested questionnaire after one-day training was given for them with their respective B.S. midwife supervisor.

Data analysis was performed using SPSS version 20.0. Variables reaching a *p* value of 0.2 on bivariate analysis were included in multiple logistic regression analysis and *p* values of less than 0.05 were taken to represent significance. The degree of association between the independent and dependent variables was analysed using odds ratios with 95% confidence intervals. Postnatal mothers who were able to mention danger signs of obstetric complications which occur during pregnancy, childbirth, and postpartum period above calculated mean were considered as knowledgeable and those who were stated below the mean were considered as not knowledgeable.

Ethical clearance was obtained from the Institutional Review Board (IRB) of University of Gondar, College of Health Sciences. A formal letter of cooperation was sent to Mechekel district health bureau and a formal letter of permission was obtained. Finally, written informed consent was obtained from each pregnant woman.

## 3. Results

### 3.1. Sociodemographic Characteristics

Four hundred five participants responded to the questionnaire, giving a response rate of 98.5%. The mean age of the study participants was 28.9 years (sd 5.63). The majority of women were married (383, 94.6%) and orthodox Christian (375, 92.6%). Two hundred thirty (56.8%) of the pregnant women had attended at least primary education (Table 1).

### 3.2. Obstetric Characteristics

For 219 (54.1%) of the mothers, the index birth was their first, while 196 (48.4%) of them had experienced pregnancy 2 to 4 times. Of the respondents, 358 (88.4%) had antenatal follow-up during their last pregnancy and 264 (65.2%) gave their last birth at health institution. Among the respondents, 140 (34.6%) have experienced obstetric complications among which obstructed/prolonged labour, 56 (13.8%), takes over the majority followed by hemorrhage, 51 (12.6%) (Table 2).

### 3.3. Knowledge about Danger Signs of Obstetric Complications

More than half, 55.1%, of study participants were knowledgeable about overall danger signs of obstetric complications that can occur during pregnancy, delivery, and postpartum/puerperium period.

Of the respondents, 211 (52.1%), 216 (53.3%), and 188 (46.4%) were stated above the calculated mean of danger signs of obstetrics complication during pregnancy, labour/delivery, and postpartum periods, respectively. On the other hand vaginal bleeding is the most frequently recalled danger sign of obstetrics complications by 244 (60.2%), 230 (56.8%), and 218 (53.8%) respondents during pregnancy, labour/delivery, and postpartum periods, respectively (Table 3).

### 3.4. Source of Information (Multiple Source Is Possible)

The most frequently reported sources of information were health service providers (including health extension workers), 309 (76.3%), followed by mass media (20.5%), friends (24.0%), and community (10.9%).

Among study participants, majority of them, 166 (41.0%), were informed about danger sign of obstetric complications at specific period of pregnancy and 97 (23.7%) were not informed about any obstetric complications during pregnancy, delivery, and postpartum or puerperium period. About 167 (41.2%) responded that they met with health extension workers by schedule to discuss their health issue and majority of them, 238 (58.8%), responded that there was no availability of health extension workers at their homes.

### 3.5. Factors Associated with Knowledge of Obstetrics Complication Danger Signs

A bivariate analysis was done to assess any association between independent variables and knowledge of obstetrics complications danger signs. After controlling the effect of other variables, family monthly income, maternal educational level, husband educational level, gravidity, ANC follow-up during last pregnancy, and place of last delivery were found to be significantly associated with missed opportunity of institutional delivery (*p* values < 0.05).

Those whose monthly family income was >1500 ETB were about 3 times more likely knowledgeable than those whose income was less than 500 ETB (AOR = 2.954, 95% CI: 1.289, 6.770). Those mothers who attended more than secondary school were about 2 times more likely knowledgeable about obstetric danger signs than those who had no schooling at all (AOR = 1.921, 95% CI: 1.004, 3.676). Similarly mothers whose husbands finished more than secondary school were about 3 times more likely knowledgeable than those whose husbands had not attended formal school (AOR = 3.163, 95% CI: 1.860, 5.3770).

Grand multipara mothers were also about 7 times more likely knowledgeable than primiparous (AOR = 7.463, 95% CI: 1.301, 12.800). Mothers who had antenatal follow-up during their last pregnancy were two times more likely knowledgeable than those who had no ANC (AOR = 2.184, 95% CI: 1.137, 4.196). In case of place of last delivery, those who gave birth to their recent infants at health institution were about two times more likely knowledgeable than those who delivered their neonates at home (1.955, 95% CI: 1.214, 3.150) (Table 4).

## 4. Discussion

The current study revealed that only 55.1% of postpartum women were knowledgeable about danger signs of obstetrics complications which is slightly consistent with research evidence from rural Tanzania (51.1%) [14] but higher than studies conducted in Uganda and Egypt [15, 16]. The reason might be due to sociocultural difference of study population and difference in health policy intervention strategy.

Maternal knowledge is still not in line with coverage of ANC follow-up in the area, like studies done in Uganda and Nigeria [16, 17]. The reason for this finding might be due to low emphasis of danger signs of obstetric complications during ANC follow-up among health service providers or due to knowledge gap of professionals and poor information offering to the mothers. The present study finding showed that mothers' knowledge was higher than the study done in rural Tanzania, specifically during pregnancy (52.1%), childbirth (53.3%), and postpartum (46.4%) period [14]. This discrepancy might be due to sociocultural difference of study population and difference in health policy intervention strategy between two countries.

Consistent with previous studies conducted in the Kwazulu-Natal province in South Africa, India, and Ethiopia [13, 18, 19], the finding of this study indicated that about 211 (52.1%) and 216 (53.3%) of respondents were knowledgeable about danger signs of obstetric complications during pregnancy and childbirth, respectively. However research evidence from Aleta Wendo, Ethiopia, indicated that about (30.4%), (41.3%), and (37.7%) were knowledgeable about obstetric danger signs during pregnancy, childbirth, and postpartum period, respectively, which was lower than the finding of this study [12]. On the other hand about quarter of respondents (24.7%) were unable to mention any danger signs of obstetric complications during pregnancy which is lower than finding from Aleta Wendo (39.0%) and Tsegedie (35.1%), Ethiopia [12, 13]. The possible reason might be the time gap between two studies, because there is progressive change in maternal health intervention strategy over time.

In line with studies done in India, Tanzania, Uganda, Nigeria, South Africa, Egypt, and Ethiopia [12–19], the finding of this study showed that there is significant association between maternal educational level and knowledge of obstetric danger sign. Postpartum mothers who had attended secondary school and above were about 2 times more likely knowledgeable than women who did not attend any school. This might be due to the fact that education is important for easily understanding health message from different sources and also it enhances their autonomy to decide on their reproductive issue and medical care utilization at any time because they are more aware of their health conditions. Similarly husband educational status was another predictor of knowledge of obstetric danger signs. Mothers whose husbands have attended secondary school and above were about 3 times more likely knowledgeable than those whose husbands have no level of education. This finding is also consistent with studies done in Guatemala and Jordan [20, 21]. The reason might be the fact that educated husbands are participant in the maternal health service utilization and decision to seek medical services compared to their counterparts.

There was statistical difference between family monthly income and women's knowledge level of danger signs of obstetric complications. Women with family monthly income of >1500 were more knowledgeable than women with <500 monthly family income. This is in line with study done in Nigeria [17]. This may be explained by the fact that income is very important to be able to visit health institution to seek medical services.

Similarly, like studies done in Egypt, Tanzania, and Ethiopia [12, 13, 15, 16], there is significant association between knowledge of obstetric danger sign and gravidity. Multiparous women were more knowledgeable than primiparous. This may be due to previous experience of pregnancy and childbirth and some of them may have experienced obstetric complications which are an important source of their information. So they were more aware of their health problems related to pregnancy, delivery, and period after delivery than primes.

ANC follow-up has significant association with mother's knowledge of obstetric danger signs. Those mothers who had ANC follow-up during their last pregnancy were about two times more likely knowledgeable than their counterparts. This is consistent with studies done in Egypt, Uganda, Tanzania, and Ethiopia [14, 16, 22, 23]. This could be due to the fact that ANC is an ideal site for the mothers to hear about obstetric danger sign especially during pregnancy. It provides an important opportunity for information, education, and communication.

In line with studies done in Tanzania and Ethiopia, there is significant statistical association between knowledge of obstetric danger sign and place of last delivery [13, 14]. Mothers who gave birth to their recent child in health institution were about two times more likely knowledgeable about danger signs of obstetric complications than those who gave birth at their homes. This might be due to the fact that when women gave birth at health institution they were advised and counseled about danger signs of obstetric complications by professionals. Another reason might be that the majority of the study participants probably attended ANC during their last pregnancy which is ideal site of health information.

## 5. Conclusion

According to the finding of this study, a significant number of mothers are still not knowledgeable about danger signs of obstetric complications.

Health service providers, including health extension workers, were the most frequently reported source of information for obstetric complications danger signs.

Maternal educational level, husband educational level, ANC follow-up during last pregnancy, family monthly income, being multiparous, and place of last delivery were factors found to be significantly associated with mothers' knowledge of obstetric danger signs.

## Figures and Tables

**Table 1:** Sociodemographic characteristics of postnatal mothers of Mechekel district health centers (*n* = 405), East Gojjam zone, Northwest Ethiopia, 2014.

Variables category	Frequency	Percentage
*Age of the mother*		
15–19	36	8.9
20–24	117	28.9
25–29	111	27.4
30–34	98	24.2
≥35	43	10.6
*Marital status of respondents*		
Married	383	94.6
Single	9	2.2
Widowed	7	1.7
Divorced	6	1.5
*Religion affiliation*		
Orthodox	375	92.6
Muslim	20	4.9
Protestant	8	2.0
Others	2	0.5
*Educational level of respondent*		
No schooling	175	43.2
Primary school	86	21.2
Secondary school	74	18.3
Above secondary school	70	17.3
*Occupation*		
Gvt/private employ	90	22.2
Housewife	173	42.7
Farmer	60	14.8
Trader	58	14.3
Others	24	5.9
*Place of residence*		
Urban	175	43.2
Rural	230	56.8
*Distance from home to health facility by car*		
One hour or less	256	63.2
Greater than an hour	149	36.8
*Participant husband educational level*		
No schooling	125	30.9
Primary school	72	17.8
Secondary school	61	15.1
Above secondary school	125	30.9
*Family monthly income*		
≤500	60	14.8
501–1000	143	35.3
1001–1500	136	33.6
≥1501	66	16.3
*Decision-makers on health service utilization*		
Self	231	57.0
With husband	112	27.7
Other persons	62	15.3

**Table 2:** Obstetric characteristic of postnatal mothers (*n* = 405) of Mechekel district health centers, East Gojjam zone, Northwest Ethiopia, 2014.

Variables category	Frequency	Percentage
*Gravidity*		
One	176	43.5
Two–four	196	48.4
Five and above	33	8.1
*Parity*		
One	219	54.1
Two–four	168	41.5
Five and above	18	4.4
*ANC during last pregnancy*		
Yes	358	88.4
No	47	11.6
*Place of ANC*		
Government hospital/HC	306	85.5
Private hospital/clinic	52	14.5
*Gestational age started ANC*		
Within 4th month and before	178	49.7
Within 4th-5th month	67	18.4
Within 6th-7th month	33	9.2
Within 8th month and after	8	2.2
Do not know	73	20.4
*Number of ANC visits*		
One time	36	10.1
Two times	75	20.9
Three times	92	25.7
Four or more	155	43.3
*Place of last delivery*		
Home	141	34.8
Health institution	264	65.2
*Mode of last delivery*		
Spontaneous vaginal delivery	297	73.3
Cesarean delivery	23	5.7
Assisted vaginal delivery	79	19.5
Others	6	1.5
*Condition of neonate at birth*		
Normal/alive	334	82.5
Abnormal/diseased	54	13.3
Dead	17	4.2
*Experience of obstetric complications*		
Yes	140	34.6
No	265	65.4
*Type of complications they had faced*		
Hemorrhage	51	12.6
PIH	31	7.7
Sepsis	12	3.0
Obstructed labour	56	13.8
Abortion	35	8.6
Others	32	7.9

**Table 3:** Proportion of recalled danger signs of obstetric complications among postnatal mothers of Mechekel district health centers, East Gojjam zone, Northwest Ethiopia, 2014 (*n* = 405).

Danger sign of obstetric complications	Proportion of mentioned danger sign during the following					
	Pregnancy		Childbirth		Postpartum period	
	*n*	%	*n*	%	*n*	%
Vaginal bleeding	218	53.8	230	56.8	244	60.2
Severe headache	212	52.3	169	41.7	120	29.6
Convulsion	122	30.1	95	23.5	89	22.0
Loss of consciousness	124	30.6	107	26.4	93	23.0
Epigastric pain	127	31.4	105	25.9	85	21.0
Blurring of vision	141	34.8	127	31.4	97	24.0
Increased/decreased fetal movement	176	43.5	137	33.8		
Fast or difficult breathing (dyspnea)	80	19.8				
Excessive vomiting	43	10.6				
Preterm labour (onset of labour before 37 weeks of gestation)	150	37.0				
Premature rupture of membrane	165	40.7				
Prolonged labour (lasting > 12 hours)			190	46.9		
Severe abdominal pain	126	31.1	91	22.5		
Retained placenta			180	44.4		
Foul-smelling vaginal discharge					156	38.5
High fever with or without abdominal pain					149	36.8
Others	44	10.9	24	5.9		
Do not know any danger sign	100	24.7	91	22.5	95	23.5

**Table 4:** Bivariate and multivariate analysis of factors associated with knowledge about danger sign of obstetric complications among postnatal mothers of Mechekel district health centers, East Gojjam zone, Northwest Ethiopia, 2014 (*n* = 405).

Variables category	Knowledge level		COR (95% CI)	AOR (95% CI)
	Yes	No		
*Place of residence*				*∗*
Urban	108 (61.7%)	67 (38.3%)	1.612 (1.081, 2.404)	
Rural	115 (50.0%)	115 (50.0%)	*1*	
*Occupation*				*∗*
Housewife	88 (50.9%)	85 (49.1%)	*1*	
Farmer	29 (48.3%)	31 (517%)	2.514 (0.993, 6.368)	
Government employ	48 (70.6%)	20 (29.4%)	5.829 (2.095, 16.215)	
Trader	37 (63.8%)	21 (36.2%)	2.272 (0.823, 6.272)	
Other	7 (29.2%)	17 (70.8%)	4.279 (1.527, 11.989)	
*Family monthly income*				
*≤*500	24 (40.0%)	36 (60.0%)	*1*	1
501–1000	74 (51.7%)	69 (48.3%)	1.609 (0.872, 2.966)	1.712 (0.866, 3.386)
1001–1500	85 (62.5%)	51 (37.5%)	2.500 (1.342, 4.658)	2.246 (1.091, 4.625)
*≥1501*	*40 (60.6%)*	*26 (39.4%)*	*2.308 (1.129, 4.715)*	*2.954 (1.289, 6.770)* ^**∗∗**^
*Maternal education*				
No schooling	79 (45.1%)	96 (54.9%)	*1*	1
Primary	48 (55.8%)	38 (44.2%)	1.535 (0.913, 2.580)	1.222 (0.674, 2.216)
Secondary	47 (63.5%)	27 (36.5%)	2.115 (1.209, 3.700)	1.921 (1.004, 3.676)
*More than secondary*	*49 (70.0%)*	*21 (30.0%)*	*2.835 (1.569, 5.123)*	*1.977 (1.052, 3.716)* ^**∗∗**^
*Distance from home to health facility*				*∗*
Less than 1 hour	152 (59.4%)	104 (40.6%)	1.606 (1.069, 2.412)	
Greater than 1 hour	71 (47.7%)	78 (52.3%)	*1*	
*Husband education*				
No schooling	51 (40.8%)	74 (59.2%)	*1*	*1*
Primary	40 (55.6%)	32 (44.4%)	0.334 (0.193, 0.545)	1.343 (0.744, 2.423)
Secondary	37 (60.7%)	24 (39.3%)	0.588 (0.324, 1.069)	1.838 (0.978, 3.453)
*More than secondary*	*85 (68.0%)*	*40 (32.0%)*	*0.725 (0.384, 1.371)*	*3.163 (1.860, 5.3770)* ^**∗∗**^
*Gravidity*				
One	103 (58.5%)	73 (41.5)	*1*	*1*
Two–four	108 (55.1%)	88 (44.9%)	2.469 (1.143, 5.333)	2.473 (0.478, 12.780)
*Five and above*	*12 (44.9%)*	*21 (63.6%)*	*2.148 (1.001, 4.607)*	*7.463 (1.301, 12.800)* ^**∗∗**^
*ANC follow-up*				
*Yes*	*207 (57.8%)*	*151 (42.2%)*	*2.656 (1.402, 5.031)*	*2.184 (1.137, 4.196)* ^**∗∗**^
No	16 (34.0%)	31 (66.0%)	1	
*GA at start of ANC*				*∗*
<4 months	65 (36.5%)	113 (63.5%)	1.516 (0.873, 2.632)	
4-5 months	39 (59.1%)	27 (40.9%)	1.259 (0.643, 2.467)	
6-7 months	14 (42.4%)	19 (57.6%)	0.642 (0.280, 1.472)	
8+ months	2 (25.0%)	6 (75.0%)	0.291 (0.055, 1.536)	
Do not know	39 (53.4%)	34 (46.6%)	*1*	
*Number of ANC visits*				*∗*
One time	19 (52.8%)	17 (47.2%)	*1*	
Two times	39 (52.0%)	36 (48.0%)	0.969 (0.437, 2.148)	
Three times	47 (51.1%)	45 (48.9%)	0.935 (0.432, 2.021)	
Four and above	102 (65.8%)	53 (34.2%)	1.722 (0.827, 3.586)	
*Place of last delivery*				
Home	61 (43.3%)	80 (56.7%)	*1*	
*Health institution*	*162 (61.4%)*	*102 (38.6%)*	*2.083 (1.375, 3.155)*	*1.955 (1.214, 3.150)* ^**∗∗**^
*Informed obstetric danger signs*				*∗*
Yes	79	87	1.669 (1.119, 2.490)	
No	95	144	*1*	

^*∗*^ *p* value < 0.2: variables found to be not significant in backward stepwise logistic regression.

^*∗∗*^ *p* value < 0.05: variables found to be significant in backward stepwise logistic regression.
